# Supplementary material for: DMRforPairs: identifying Differentially Methylated Regions between unique samples using array based methylation profiles
Source: BMC Bioinformatics. 2014 May 15;15:141. doi: 10.1186/1471-2105-15-141 (PMC4046028; doi:10.1186/1471-2105-15-141)
Supplement: Additional file 2 — DMRforPairs output for the comparison of A431-MCF7 and NA17018-NA17105. Please start from the HTML files in each folder. Available via the BMC Bioinformatics website. [file 1471-2105-15-141-S2.zip › 1394847754114233_MOESM2_ESM/A431_MCF7/figures/20390.pdf]

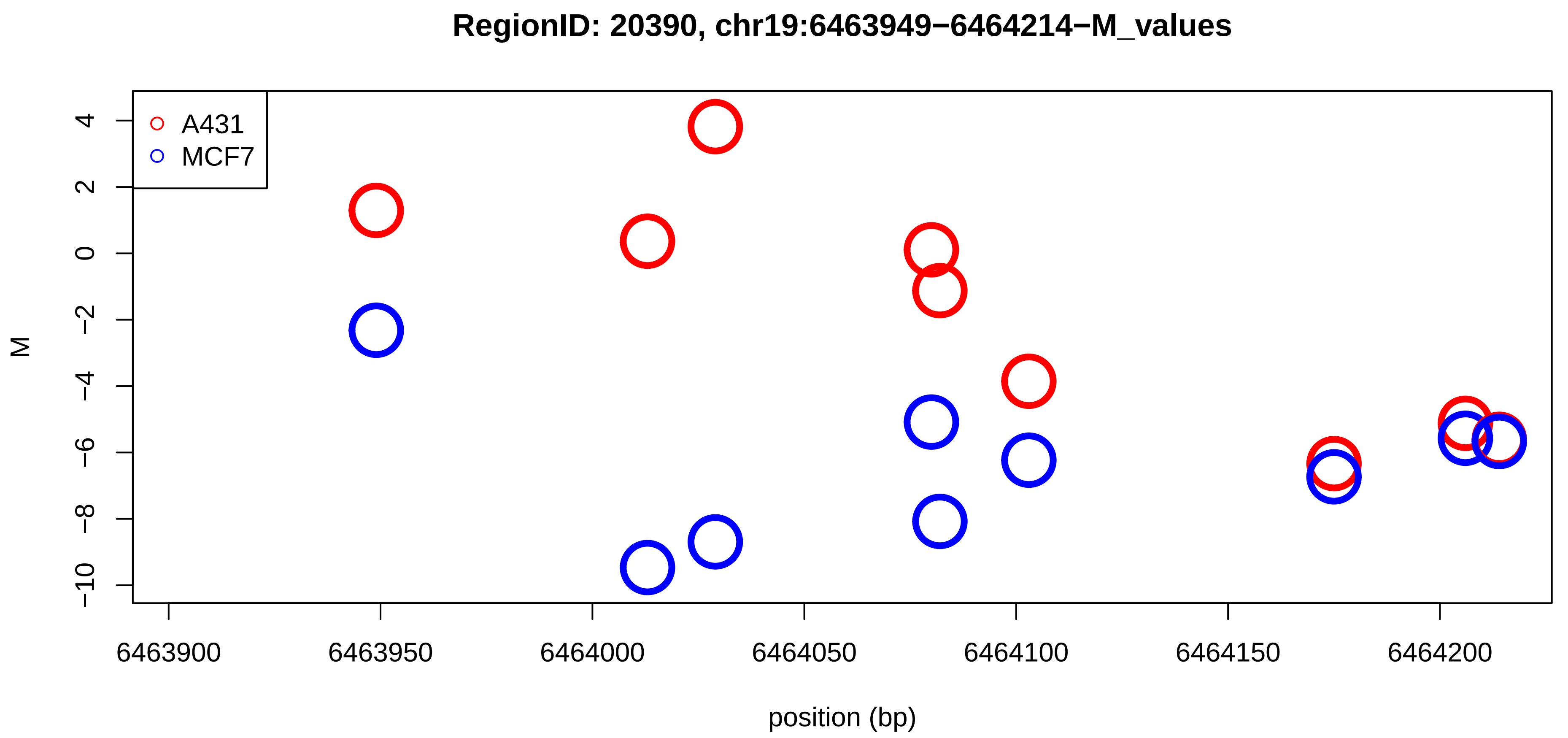
RegionID: 20390, chr19:6463949−6464214−M_values
A431
MCF7
M
4
2
0
−2
−4
−6
−8
−10
6463900
6463950
6464000
6464050
6464100
6464150
6464200
position (bp)

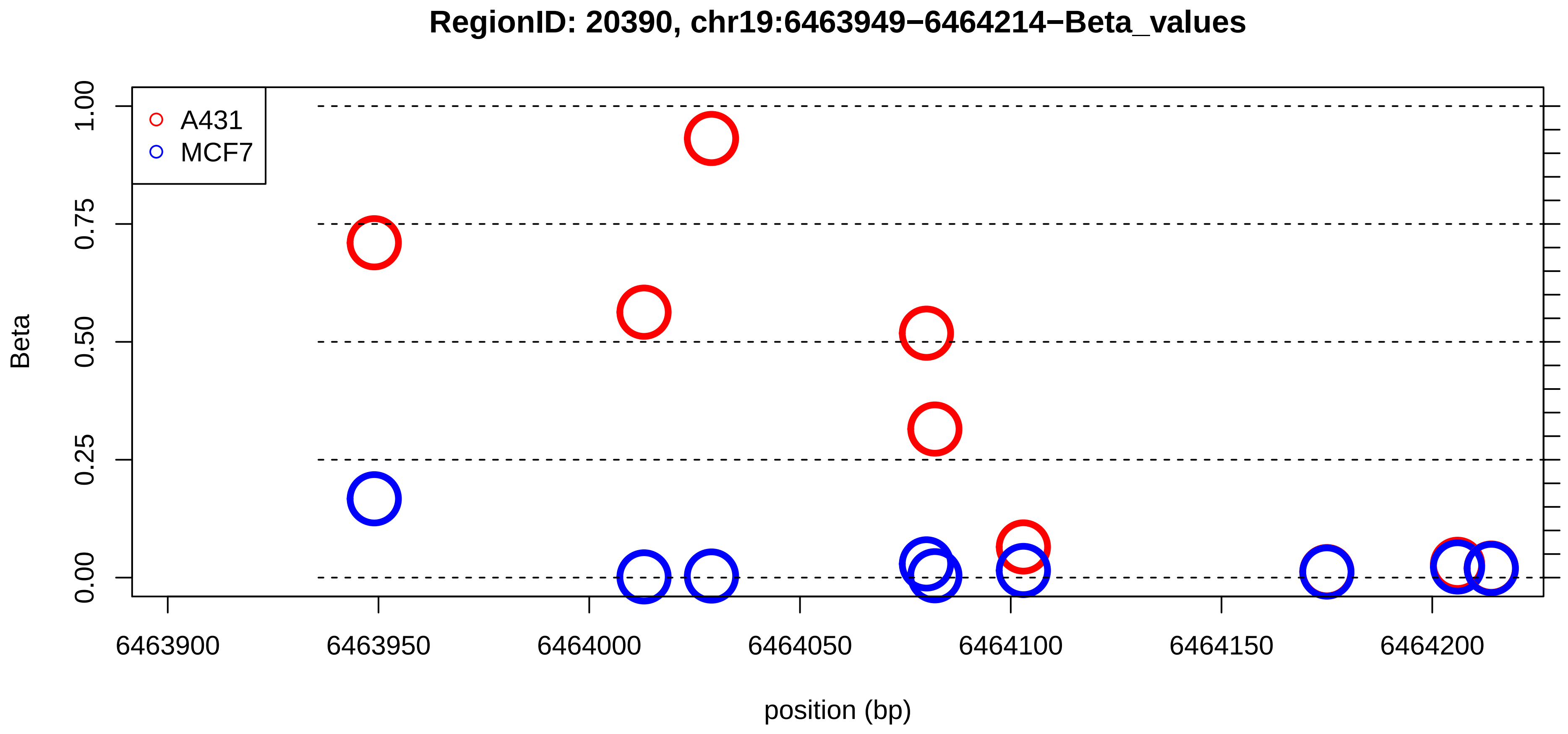
RegionID: 20390, chr19:6463949−6464214−Beta_values
A431
MCF7
Beta
1.00
0.75
0.50
0.25
0.00
6463900
6463950
6464000
6464050
6464100
6464150
6464200
position (bp)

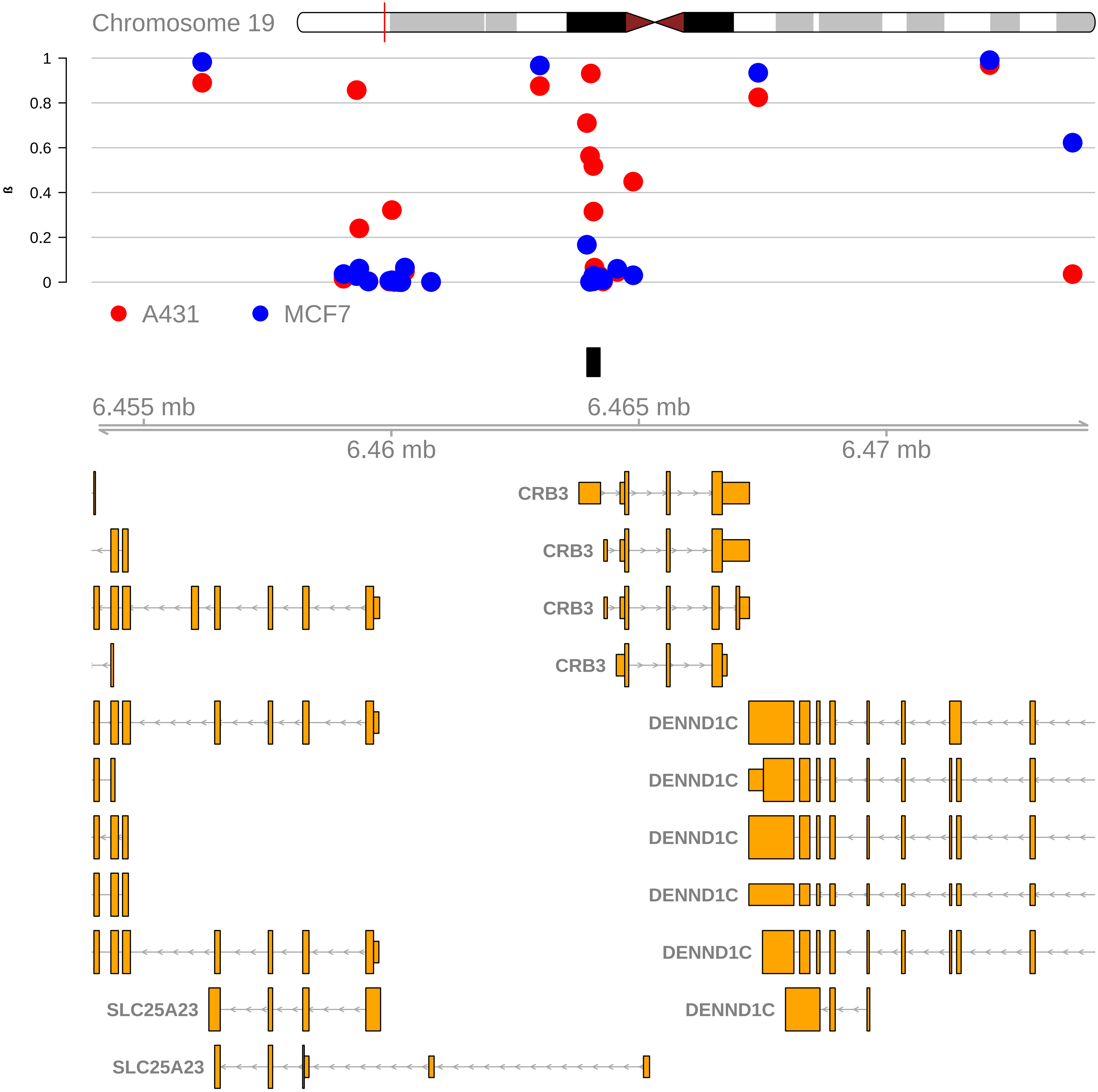
Chromosome 19
ß
1
0.8
0.6
0.4
0.2
0
A431
MCF7
6.455 mb
6.465 mb
6.46 mb
6.47 mb
CRB3
CRB3
CRB3
CRB3
DENND1C
DENND1C
DENND1C
DENND1C
DENND1C
DENND1C
SLC25A23
SLC25A23
